# Exome sequencing reveals broad genetic heterogeneity for neuromuscular disorders in consanguineous Pakistani Families

**DOI:** 10.1038/s41431-025-01915-9

**Published:** 2025-07-29

**Authors:** Tooba Aleem, Maliha Rashid, Naeem Ahmad, Muhammad Farrukh Asif, Muhammad Tariq, Naveed Altaf Malik, James A. Poulter

**Affiliations:** 1 National Institute for Biotechnology and Genetic Engineering College, Pakistan Institute of Engineering and Applied Sciences (NIBGE-C, PIEAS), Faisalabad, Pakistan; 2 Division of Molecular Medicine, Leeds Institute of Medical Research, University of Leeds, Leeds, UK

**Keywords:** Medical genetics, Genetics research

## Abstract

Neuromuscular disorders comprise the majority of neurogenetic conditions, generally characterized by overlapping clinical symptoms, such as spastic paraplegia, muscular abnormalities, and ataxia. In low- and middle-income countries (LMICs), many patients remain undiagnosed or are misdiagnosed. For many NMDs, early diagnosis helps reduce the impact and mortality of the disorder, particularly in LMICs such as Pakistan, and reduces the burden on the healthcare system. The aim of this study was to use exome sequencing as a first line of diagnostic approach to identify the cause of disease. Here, we present five consanguineous families from different remote villages in Pakistan with an undiagnosed neuromuscular disorder, in whom whole-exome sequencing was able to provide a diagnosis. We identified novel variants in known reported disease genes *SPEN* (c.351_356del) and *POMT1* (c.1583A > G) and three previously reported variants in *MMP2* (c.1287del), *ARL13B* (c.599 G > A), and *SPG11* (c.6811_6812del). In one family, homozygous pathogenic variants in two different genes (*SPEN* and *NPHP4*) were identified; to our knowledge, this is the first report of nephronophthisis and Radio-Tartaglia syndrome co- segregating in a family. In all cases, Sanger sequencing was performed on available family members to confirm segregation. Our study highlights the importance of whole-exome sequencing as a first-line diagnostic approach in undiagnosed individuals with neuromuscular disorders in LMICs, where access to healthcare is limited.

## Introduction

Neuromuscular disorders (NMDs) are a diverse group of conditions that are a major cause of disability and/or mortality worldwide [1]. Classification of NMDs is challenging and is highly dependent upon the organ that is primarily affected and their severity. In most cases of NMD, a combination of the motor neurons, neuromuscular junctions, peripheral nerves, and muscles are affected [2, 3]. Additionally, the phenotype varies depending on a range of factors, including environmental factors, penetrance, or background genetic variation. This means affected individuals from the same family, with the same pathogenic variants, can have differing phenotypes that may appear neurological, or may have an appearance more in keeping with a myopathy [4]. In some cases, a single patient may appear to have multiple phenotypes, or even two different conditions [5, 6]. While individually rare, collectively NMDs represent a significant group of disorders [7], with an overall yearly incidence rate reported to be 122/100,000 people [8]. In countries with a higher incidence of consanguinity and autosomal recessive disease, the prevalence of NMDs is likely much higher. For many such countries, no epidemiologic data are currently available, although current estimates suggest a pooled prevalence of neurodevelopmental disorders across Asia to be 7.6 per 1000 [9].

To date, more than 600 genes have been identified as a cause of NMD [10], and the number continues to grow [8, 11]. Despite many patients being affected, prognostic information regarding many rare NMDs is lacking [12, 13], which impacts clinical care and treatment. For a number of neuromuscular disorders, potential therapies are currently in development or are undergoing clinical trials, but few have yet received approval for clinical use [14]. In contrast, for some NMDs, the prognosis is good with long-term medication and/or lifestyle modification [15], provided a correct diagnosis has been made and the medication is available to the patient.

The traditional approach used to diagnose NMDs includes clinical phenotyping alongside biochemical tests and tissue phenotyping such as muscle imaging, electromyogram, muscle biopsy, and potentially Sanger sequencing of a single or panel of genes. The accurate diagnosis of these disorders, however, is often hampered by the large diversity in clinical phenotypes [16]. More recently, whole-exome sequencing (WES) and whole-genome sequencing (WGS) have supported clinical phenotyping, increasing the diagnostic yield from 20% to 70% when performed alongside accurate phenotyping [17, 18]. WES and WGS offer rapid, cost-efficient, and precise molecular diagnosis in patients with complex, unsolved disorders affecting the peripheral and central nervous system [19, 20]. In patients with recessive conditions and consanguineous parents, regions of homozygosity frequently contain the pathogenic variants [21], allowing homozygosity mapping to further reduce the number of genomic loci to investigate.

Understanding the genetic basis for an NMD is crucial. It provides an accurate genetic diagnosis, identifies possible alternative treatment options, and informs family planning. While the importance of genetic investigations cannot be understated, genetic testing is often limited to those in developed countries. In low- and middle-income countries, such as Pakistan, with a population of nearly 250 million people, with a birth rate of 3.9 per women. Pakistan is one of the most genetically diverse countries of Asia due to its complex population history and location at the crossroads of central, western, and southern Asia. Historical migration and their mixing with local community have made its major ethnic groups further divided into subgroups. Despite this diversity, consanguineous marriages are common among first and second cousins. The rate of consanguinity in Pakistan is approaching 70%, leading to a higher prevalence of recessive genetic disorders [22]. This is further complicated by a lack of access to hospitals and expertise to manage and treat rare disorders, and due to a lack of infrastructure, most patients are unable to get a diagnosis or treatment. In Pakistan, most patients remain without a diagnosis, however, the true scale of this problem is unknown.

Here, we present five unrelated families from remote villages in Pakistan in whom multiple family members were affected with an autosomal recessive undiagnosed NMD. We demonstrate the utility of whole exome sequencing and homozygosity mapping as a first-stage diagnostic approach in these families. In each case, pathogenic variants in known NMD genes were identified, including previously unreported variants in *POMT1, NPHP4*, and *SPEN*, and three previously reported variants in *MMP2*, *ARL13B*, and *SPG11*.

## Materials and methods

### Ethical considerations and sample collection

This study was conducted following approval from the Institutional Review Board and Ethical Committee at the National Institute for Biotechnology and Genetic Engineering (NIBGE), Faisalabad, Pakistan, and by the Yorkshire and Humber—Leeds East Research Ethics Committee (REC ref. 18/YH/0070). Families were collected through field-based sampling in collaboration with local contacts and community engagement. Families were selected based on them having a suspected neuromuscular disorder. The geographical distribution of families is shown in Supplementary Fig. 1. Written and informed consent was obtained from the family head or guardian to be part of this study and to be included in any publication. All procedures adhered to the tenets outlined in the Declaration of Helsinki. Additional consent was obtained for the publication of photographs. Clinical information was gathered for each affected individual via direct interview with the patient or family members, alongside a physical assessment by the research team at the time of recruitment. Due to their rural location, clinical investigations (e.g., biochemical tests) were not performed for most of the families, however, where possible, basic tests (e.g. blood counts) were conducted when necessary. Peripheral blood samples were collected in EDTA-containing vacutainers from all affected and healthy individuals. DNA extraction was performed using the GeneJET Genomic DNA Purification Kit (Thermo Fisher Scientific).

### Whole-exome sequencing, variant filtration, and in silico analysis

Whole-exome sequencing was performed at the Next Generation Sequencing Facility, University of Leeds, UK. Genomic DNA libraries from two affected individuals per family were prepared using a Twist Exome 2.0 kit and sequenced on a NextSeq2000 P3 (300 cycle) using a 2 × 150 bp paired-end cycle. The resulting fastq files underwent quality control and adapter trimming using FastQC and cutadapt, respectively. Sequences were aligned to the GRCh38 Human reference genome using BWA-MEM and processed following the GATK best practice guidelines [23]. Variants were called using the GATK HaplotypeCaller tool and annotated with variant effector predictor incorporating CADD v1.7, SpliceAI, and gnomAD. Integrated Genome Viewer (IGV) was used to visualize and verify rare nonsynonymous and splice-site variants. Agile MultiIdeogram was used to identify regions of homozygosity from unfiltered exome VCF files (https://www.dna-leeds.co.uk/agile/AgileMultiIdeogram/). While chromosome X was not analyzed for regions of homozygosity, variants on the X chromosome were investigated in families where an X-linked mode of inheritance could not be excluded. Variant filtering and segregation were performed using VASE (https://github.com/david-a-parry/vase).

Candidate variants in genes associated with neuromuscular or neurodevelopmental disorders were prioritized, and pathogenicity prediction scores of the variants were checked using different in silico tools, including PolyPhen2, SIFT, and CADD v1.7 [24, 25]. Genotype-phenotype correlation and potential clinical significance were checked using publicly available databases, including ClinVar and Human Gene Mutation Database (HGMD). Variants were also assessed according to the American College of Medical Genetics and Genomics (ACMG) guidelines using Franklin (https://franklin.genoox.com). After assessing variants, remaining candidates were confirmed and segregated in all available family members by Sanger sequencing. Primers were designed using Primer3 (https://primer3.ut.ee/B44) (Supplementary Table 1). Electropherograms from Sanger sequencing were analyzed using Sequencher 5.0. The variants considered disease-causing have been submitted to ClinVar (submission ID: SUB15001128).

## Results

**Fig. 1:**
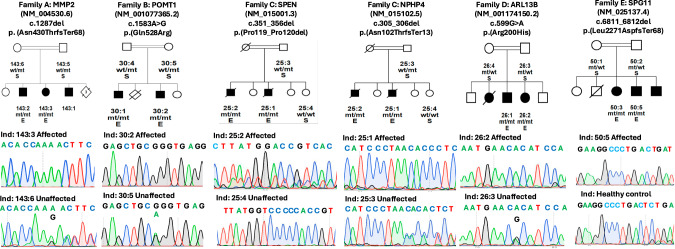
Summary of five Families (A–E) presented in this study. Families were sampled from different remote villages of Pakistan. The pedigrees, segregation, and chromatogram of each pathogenic variant identified are shown. E (exome sequenced), S (Sanger sequenced), mt (mutant allele), wt (wild type allele), ind (individual).

We identified five consanguineous families (A-E), residing in rural villages of Pakistan, in whom multiple family members were affected with an undiagnosed NMD (Fig. 1). Following whole-exome sequencing, rare homozygous variants within shared regions of homozygosity were investigated.

### Family A

Family A (HD143) presented with three affected siblings with a severe NMD, characterized by coarse facial features and skeletal abnormalities (Supplementary Table 2). The family also reported frequent miscarriages in the 1st gestational period. The male proband (143:1), aged 6-years at the time of examination, was delivered at full term without any complications during pregnancy. He achieved all developmental milestones on time, but at the time of examination, he exhibited an abnormal gait, scoliosis, an inability to fully extend his fingers, and thickened skin around the wrists and ankles. He also had a ventricular septal defect (VSD) with mild pulmonary stenosis, which was not observed in other affected family members. The probands’ two affected siblings (143:2 and 143:3), who were both older, had similar phenotypes but had progressed with age to a more severe phenotype. In particular, the eldest female sibling (143:3), aged 10 years at examination, was completely bedridden. She had joint contractures in her elbows, fingers, and toes, along with foot deformities. All the affected siblings had a history of recurrent respiratory infections. There was no known history of epileptic seizures in any of the affected siblings.

Whole-exome sequencing of two affected siblings (143:2 and 143:3) identified three missense variants and one frameshift variant (Supplementary Table 3). The homozygous frameshift variant (NM_004530.6:c.1287del; p.(Asn430ThrfsTer68)) in *MMP2* is within a homozygous region on chromosome 1 shared by affected siblings (Fig. 1). The variant is rare in gnomAD v4.0.0, with a minor allele frequency of 0.000003101 (with no homozygous individuals) and an allele frequency of 0.00005494 in the South Asian population. Pathogenic variants in *MMP2* are a known cause of autosomal recessive Multicentric osteolysis nodulosis and arthropathy (MONA) (OMIM #259600), which has overlapping phenotypes of affected individuals in Family A, such as flexion contractures and widening of metacarpals and metatarsals. According to ACMG classification, this variant is Pathogenic (Table 1), is present in ClinVar (VCV000198809.9), and was previously reported as pathogenic [26]. Sanger sequencing confirmed the variant segregated in all available family members.

**Table 1:** Pathogenic variants identified in each family in this study.

Family	Gene			ACMG classification				
		Putative function	Variants (HGVS)	ACMG class	ACMG criteria	CADD Phred score	South Asian Allele frequency	Reference (if previously published)
**A**	*MMP2* NM_004530.6	Frameshift	c.1287del; p.(Asn430ThrfsTer68)	Pathogenic	PM3 PVS1 PM2 PP5	34	0.00005494	PMID:26601801
**B**	*POMT1* NM_001077365.2	Missense	c.1583A > G; p.(Gln528Arg)	Uncertain significance	PP3 PM2	32	N/A	This study
**C**	*SPEN* NM_015001.3	In frame deletion	c.351_356del; p.(Pro119_Pro120del)	Uncertain significance	PP3 PS3 PM2	22	0.0001208	This study
	*NPHP4* NM_015102.5	Frameshift	c.305_306del; p.(Asn102ThrfsTer13)	Likely Pathogenic	PVS1 PM2	27	0.00003481	This study
**D**	*ARL13B* NM_001174150.2	Missense	c.599G > A; p.(Arg200His)	Likely pathogenic	PM2 PP3 PM5 PP5	33	0.00002196	PMID:29255182
**E**	*SPG11* NM_025137.4	Frameshift	c.6811_6812del; p.(Leu2271AspfsTer68)	Pathogenic	PM3 PVS1 PM2 PP5	34	N/A	PMID:24833714

CADD scores were calculated using CADD v.1.7. The South Asian allele frequency was obtained from gnomAD v4.0.

### Family B

Family B (HD-30) presented with two affected siblings aged 5 and 2.5 years, respectively (at last clinical assessment), exhibiting similar features including drooling, delayed developmental milestones, and muscle contracture in all limbs. They could only walk with the help of support. Both individuals were delivered by C-section. There is no history of epileptic seizures in either affected individual (Supplementary Table 2).

Both affected siblings (30:1 and 30:2) were exome sequenced and analyzed for rare homozygous variants in shared homozygous regions. This revealed a previously unreported homozygous missense variant (NM_001077365.2:c.1583A > G; p.(Gln528Arg) in *POMT1*, which is present in a shared homozygous region on chromosome 9 (Fig. 1). According to ACMG classification, this is a variant of uncertain significance (VUS). Pathogenic variants in *POMT1* are a known cause of Muscular dystrophy-dystroglycanopathy (limb-girdle), type C, 1 (OMIM# 609308), which is consistent with the phenotype of affected individuals in this family. This variant is not present in gnomAD v4.0.0 nor in ClinVar and has a CADD score of 32.0 (Table 1). Sanger sequencing confirmed the variant segregated in all affected individuals. This variant has not been reported previously as a cause of disease.

### Family C

This family (HD-25) presented with two siblings affected with a congenital neuromuscular disorder with unilateral tremors on the right side of the body. The affected individuals had global developmental delays and a history of epileptic seizures without any evidence of intellectual disability. Both affected siblings also required blood transfusions due to poor renal function (Supplementary Table 2). The proband (25:1, male), aged 15 years at the time of examination, was delivered at full term without any complications during pregnancy. He had a history of foaming seizures, which were controlled with Epival. He displayed global developmental delay and developed muscle contracture and degeneration in a progressive manner, becoming bedridden and requiring basic life care. At the time of assessment, this individual did not have any renal complications but was severely anemic, requiring 9 bottles of blood to be transfused in a year. At age 16 years, he developed renal failure with high levels of blood serum creatinine (8.3 mg/dl). The proband’s brother, (25:2), aged 11 years old at the time of examination, was also delivered at full term (vaginal delivery) with no pregnancy complications. His disease course followed that of his brother, but he developed renal failure at the earlier age of 11, with a blood creatinine concentration of 13 mg/dl. Both children died aged 16 and 12, respectively, due to renal failure.

Whole-exome sequencing of both affected siblings identified four missense variants, two in-frame deletions, and one frameshift variant (Supplementary Table 5). Homozygosity mapping identified homozygous regions shared by both affected individuals on chromosomes 1, 6, 8, 9, and 17 (Supplementary Fig. 2). A homozygous in-frame deletion (NM_015001.3:c.351_356del; p.(Pro119_Pro120del) in *SPEN* was identified as the likely cause of disease. This variant is present in gnomAD v4.0.0 with a minor allele frequency of 0.000006815 but is more common in the South Asian population with an allele frequency of 0.0001208 (11/91,084 alleles). The variant is not present in ClinVar, nor has it been previously associated with disease. According to ACMG classification, this variant is classified as a variant of uncertain significance (Table 1). Pathogenic variants in *SPEN* are associated with Radia-Tartaglia Syndrome (OMIM#613484), which shows significant phenotypic overlap with the affected individuals in Family B. Sanger sequencing confirmed the variant segregated in the family. Within the same region of homozygosity, we also identified a homozygous frameshift variant in *NPHP4* (NM_015102.5:c.305_306del; p.(Asn102ThrfsTer13)) (Supplementary Table 5) that co-segregated with the *SPEN* variant. Pathogenic *NPHP4* variants are a known cause of Nephronophthisis, which is likely to account for the renal failure observed. Sanger sequencing confirmed the *NPHP4* variant segregated in the family. According to ACMG classification, this variant is classified as Likely Pathogenic.

### Family D

This family (HD-26) presented with two siblings with severe congenital muscle contracture with ataxic gait, facial dysmorphism, strabismus, and intellectual disability (Supplementary Table 2). The proband (26:1, male), aged 22 years at the time of examination, was delivered at full term without any complications, but developed seizures ~7 days after birth. He showed delayed developmental milestones, developed muscle contractures to the point he now cannot walk. He is dependent for basic life care and feeding. Both affected patients displayed mild intellectual disability with an aggressive nature. The proband’s sister (26:2, female), aged 11 years old at the time of examination, was also delivered at full term with no pregnancy complications. She displayed the same phenotype as the proband, but without seizures, and had a more severe presentation in terms of facial dysmorphism and intellectual disability.

Whole exome sequencing of both affected siblings identified three missense variants (Supplementary Table 6), including a homozygous variant in *ARL13B* (NM_001174150.2:c.599G > A; p.(Arg200His)), which lies within the largest shared homozygous region (chr3:81594016-125470642) (Supplementary Fig. 2). This variant is classified as Pathogenic/Likely Pathogenic in ClinVar (VCV000266096.33), and rare in gnomAD with an allele frequency of 0.000008177, with no reported homozygotes, and is completely absent in the South Asian population. According to the ACMG classification, this variant is Likely Pathogenic (Table 1). Biallelic pathogenic variants in *ARL13B* are a known cause of Joubert Syndrome 8 (OMIM# 612291), which has a phenotype that overlaps with the affected individuals in this family. Furthermore, this variant has previously been reported as pathogenic [27, 28]. Sanger sequencing confirmed the variant segregated in all available family members, confirming this to be the likely cause of disease in this Family.

### Family E

This family (HD-50) presented with three affected siblings exhibiting similar features, including facial dysmorphism, delayed developmental milestones, and a progressive deterioration of walking ability (Supplementary Table 2). The onset of ambulation difficulty occurred between 10 and 13 years of age in all siblings, characterized by an ataxic gait and poor balance. There was no history of epileptic seizures in any of the affected individuals.

Whole-exome sequencing of two affected siblings (50:03 and 50:05) identified three missense variants and one frameshift variant that were homozygous in shared regions of homozygosity (Supplementary Table 7). A frameshift variant in *SPG11* (NM_025137.4:c.6811_6812del; p.(Leu2271AspfsTer68) was identified in the largest homozygous region on chromosome 15. This variant is classified as Pathogenic in ClinVar (VCV001180685.5) but is not present in gnomAD. According to ACMG classification, this variant is Pathogenic. Pathogenic variants in *SPG11* are associated with spastic paraplegia with mild intellectual disability (OMIM #604360), which is in keeping with the phenotype of affected individuals in this family. Furthermore, this variant has been previously reported as pathogenic [29]. Sanger sequencing confirmed the variant segregated in all available family members.

## Discussion

Understanding the pathophysiology of NMDs ultimately helps in disease management, accurate molecular diagnosis, prenatal screening, and genetic counseling for recurrence risk for future pregnancies, as well as helping guide development of potential therapeutics [30]. Additionally, in lower- and middle-income countries, such as Pakistan, where all families in this study reside, a conclusive molecular diagnosis may help in preventing excessive testing, reduce the burden on the health care system and parents, improve quality of life by providing psychological benefits, and offer prognostic information [31]. Despite breakthroughs in genetic testing techniques such as WES and homozygosity mapping, pinpointing pathogenic variants is challenging for neurodevelopmental and neuromuscular disorders due to genetic and clinical heterogeneity. More than 50% of affected individuals often do not receive a genetic diagnosis, receive multiple diagnoses, or face misdiagnosis, which later contributes to a prolonged and difficult diagnostic odyssey [32].

According to recommendations by ACMG, next generation sequencing should be the first or second line of testing for patients with congenital anomalies such as Intellectual Disability (ID) and NMD [33, 34]. Due to its quick, financially viable, and precise diagnosis for affected individuals with overlapping, uncharacterized, heterogeneous NMDs, whole-exome sequencing has become the standard diagnostic tool, achieving a diagnostic success rate of ~30–50% [20]. In this study, we further demonstrated the utility of whole-exome sequencing in NMDs by sequencing five undiagnosed families in whom no prior sequencing had been performed.

In three families, we identified previously reported pathogenic variants in known NMD genes. In Family A, we identified a frameshift variant, p.(Asn430ThrfsTer68), in matrix metalloproteinase-2 (MMP2), an enzyme involved in the degradation of extracellular matrix components and plays a vital role in cell attachment and apoptosis [35]. The variant lies within the catalytic domain; however is predicted to undergo nonsense-mediated mRNA decay, leading to an MMP2 deficiency [26]. In Family D, we identified a recurrent likely pathogenic missense variant in ARL13B (p.(Arg200His)), encoding an ADP ribosylation factor-like (ARL), small GTPase, which is part of the RAS superfamily acting as a molecular switcher. Unlike other members of this family, ARL13B lacks a key glutamine residue needed for intrinsic GTPase activity, which is crucial for cellular signaling [36]. The affected female in this family had a more severe presentation of ataxia, which is a phenomenon previously reported, possibly due to increased *ARL13B* expression in the female brain compared to males [27]. In Family E, a homozygous frameshift deletion (p.(Leu2271AspfsTer68)) was identified in SPG11, reported previously to result in a premature stop codon [37]. *SPG11* encodes spatacsin, which plays a crucial role in the maintenance and growth of neuronal axons and the regulation of intracellular cargo movement [38].

In the remaining two families, novel variants in associated disease genes were identified. In Family B, an unreported variant in POMT1 p.(Gln528Arg) was identified. POMT1 is an enzyme involved in post-translational modification through its O-mannosyltransferase activity [39]. It binds with specific substrates and catalyzes the transfer of mannose sugar from dolichol-phosphate-mannose (Dol-P-Man) to serine/threonine on target protein, such as alpha-dystroglycan (α-DG) [40]. This modification is crucial for the functional and structural integrity of α-DG and other proteins in the brain and muscles [41]. Our identified missense was found in the Protein O-mannosyl-transferase 1 domain, which may impact its functional role.

In Family C, we identified a previously unreported in-frame deletion in *SPEN*, which is associated with Radio-Tartaglia syndrome (RATARS). Interestingly, published cases of RATARS have an autosomal dominant mode of inheritance, but in this study, only homozygous individuals displayed a phenotype [42]. *SPEN* encodes a 3664 amino acid transcriptional repressor protein in which all published cases of RATARS are heterozygous protein truncation variants located between Arg535 and Asn3652, resulting in haploinsufficiency of SPEN. In contrast, in Family C, we found an in-frame 2 amino acid deletion at the N-terminal region of the protein, resulting in the deletion of Pro119 and Pro120, which is unlikely to impact protein expression. Important domains in SPEN include a nuclear localization domain, RNA recognition motif (RRMs), and nuclear receptor interaction domain (RID) [43]. Notably, our in-frame deletion variant is in the DNA-binding domain, possibly leading to reduced DNA binding and subsequently a reduction in transcriptional repression. This difference in the underlying disease mechanism may explain why a phenotype is only observed in homozygous individuals in this study and suggests hypomorphic biallelic *SPEN* variants may also underly RATARS, mimicking those due to heterozygous truncating variants. This is supported by a recent study reporting SPEN loss of function leads to severe developmental defects, including brain abnormalities in mice [42]. Both affected patients were anemic, requiring frequent blood transfusions, a phenotype not reported in previous literature associated with RATARS. Instead, within the same shared homozygous region as *SPEN*, we identified a homozygous 2 bp deletion in *NPHP4* resulting in a frameshift and premature termination codon. Retrospective investigations revealed both affected siblings had high blood serum creatinine and urea concentrations. Pathogenic *NPHP4* variants cause nephronophthisis type 4, in which early-stage kidney dysfunction, or even kidney failure, is observed [44]. It is therefore likely that the affected individuals in this family have two distinct disorders. To our knowledge, these are the first cases reported with nephronophthisis and Radio-Tartaglia syndrome.

Altogether, this study demonstrates how whole-exome sequencing of families in understudied consanguineous populations can lead to the discovery of novel rare variants, modes of inheritance, and give insight in variant impact and gene function. We therefore emphasize the importance of whole-exome sequencing as a first-pass test for NMDs in LMICs for affected families and for clinical practice. For example, the identification of pathogenic variants in genes such as *NPHP4* helps in timely intervention, such as routine kidney testing for high-risk families, and informing reproductive genetic testing via carrier screening. Yet, a few limitations remain. Exome sequencing is not able to detect deep intronic variants, and copy number analysis remains challenging, leaving many genomic variants undiscovered. Genome sequencing may overcome these challenges [45], but the cost and increased computational requirements mean they are currently not suitable as a first-pass test for most LMICs. Additionally, variant interpretation is hampered by the limited variant information available for South Asian populations in population databases, such as gnomAD and ClinVar, which may result in misinterpretation of variants. In a recent study, the South Asian population was found to have the biggest variant knowledge deficit, double that of other populations (43.4% of private variants were not reported in ClinVar compared to 20–30% for all other populations) [46]. Initiatives to improve access to sequencing for under-represented and diverse populations, and for this data to be ethically deposited into public databases such as ClinVar and gnomAD, are therefore urgently needed to improve variant interpretation.

Despite the high burden of genetic disease, driven in some part by high rates of consanguinity, there are few genetic studies characterizing the spectrum and frequency of clinically relevant genetic variants in Pakistan, particularly in rural areas where access to medical services is limited. This lack of information provides challenges in developing a healthcare system that meets the needs of the local population. Moreover, if common founder variants are identified, screening programs can be initiated to facilitate quick diagnoses and inform future care. In our study, we did not find any recurrent or shared variants among the families analyzed, suggesting there is genetic heterogeneity underlying NMDs in Pakistan and not a common underlying cause. This diversity highlights the value of WES as an unbiased method to comprehensively study genes simultaneously, unlike targeted single-gene analyses traditionally used for disorders such as β-thalassemia.

In conclusion, we report the results of whole sequencing of five families with an inherited neuromuscular disease. In two families, novel variants in already reported NMD genes were identified, and in the remaining three families, previously reported pathogenic variants in *MMP2*, *ARL13B*, and *SPG11* were identified. The identification of these variants has direct translational value to the families for carrier screening, facilitating ongoing clinical management, and will facilitate informed decisions to be made for future marriages and pregnancies to reduce the risk of further children being born with an NMD.

## Supplementary information

Supplementary Data

## Data Availability

The variants reported in this study have been submitted to ClinVar (accession numbers: SCV005684963-SCV005684968).
