# Supplementary material for: Exome sequencing reveals broad genetic heterogeneity for neuromuscular disorders in consanguineous Pakistani Families
Source: Eur J Hum Genet. 2025 Jul 29;33(10):1264–70. doi: 10.1038/s41431-025-01915-9 (PMC12480867; doi:10.1038/s41431-025-01915-9)
Supplement: Supplementary file 1 — Supplementary Data [file 41431_2025_1915_MOESM1_ESM.docx]

**Supplementary Data**

**Exome sequencing reveals broad genetic heterogeneity for neuromuscular disorders in consanguineous Pakistani Families.**

Tooba Aleem^1, 2^, Maliha Rashid^1^, Naeem Ahmad^1^, Muhammad Farrukh Asif^1^, Muhammad Tariq^1^, Naveed Altaf Malik^1^, James A. Poulter^2^.

^1^National Institute for Biotechnology & Genetic Engineering College Pakistan Institute of Engineering & Applied Sciences (NIBGE-C, PIEAS) Jhang Road, Faisalabad

^2^Division of Molecular Medicine, Leeds Institute of Medical Research, University of Leeds, Leeds, UK

**Supplementary Table 1:** Sequences of primers used to confirm and segregate candidate variants.

**Supplementary Table 2:** Clinical summary of affected individuals across all families in this study.

**Supplementary Table 3:** List of variants remaining after filtering (Family A)

**Supplementary Table 4:** List of variants remaining after filtering (Family B)

**Supplementary Table 5:** List of variants remaining after filtering (Family C)

**Supplementary Table 6:** List of variants remaining after filtering (Family D)

**Supplementary Table 7:** List of variants remaining after filtering (Family E)

**Supplementary Figure 1:** Map showing the location of five families sampled from different region of Pakistan

**Supplementary Figure 2:** Ideograms showing regions of homozygosity shared between affected individuals with exome data for each Family

**Supplementary Table 1: Sequences of primers used to confirm and segregate candidate variants.**

| Family | Gene | Forward Primer (5’-3’) | Reverse Primer (5’-3’) |
| --- | --- | --- | --- |
| A | *MMP2* | GTACAGCCTGTTCCTCGTGG | AGCTCCTGAATGCCCTTGAT |
| B | *POMT1* | GCCCTTGTCTGTTCTGCCAG | AGTCATCACTTCTCAGCGC |
| C | *SPEN* | AATGAACCAGGCACCATCCC | GTCTCCGCTCATAACGTCCT |
| C | *NPHP4* | GCCCATGTCCTCTTTGTGTG | TTTGTCCTGGGAAGCAGAGA |
| D | *ARL13B* | CATGTTCAGCAATCTCGGGG | CGCACTCGTTCAGCTCTTTC |
| E | *SPG11* | CAAATCTTCTTTATTTCCCC | GAAAAAAGCCTTTGGGTTAC |
|  |  |  |  |

**Supplementary Table 2: Clinical summary of affected individuals across all families described in this study.**

|  | **143:2** | **143:3** | **143:1** | **30:1** | **30:2** | **26:1** | **26:2** | **25:1** | **25:2** | **50:3** | **50:3** |  |  |
| --- | --- | --- | --- | --- | --- | --- | --- | --- | --- | --- | --- | --- | --- |
| **Age (years) at examination** | 6 | 10 | 8 | 5 | 2.5 | 22 | 11 | 15 | 11 | 18 | 21 |  |  |
| **Disease Onset** | Unknown | Unknown | Unknown | Congenital | Congenital | Congenital | Congenital | Progressive | Progressive | Progressive | Progressive |  |  |
| **Gender** | Male | Female | Male | Male | Male | Male | Female | Male | Male | Female | Male |  |  |
| **Facial**  **Dimorphism** | + | + | + | - | - | + | + | - | - | + | + |  |  |
| **Developmental milestone** | Achieved on Time | Achieved on Time | Achieved on Time | Delayed | Delayed | Delayed | Delayed | Delayed | Delayed | Delayed | Delayed |  |  |
| **Muscles contracture** | + | + | + | + | **+** | **+** | **+** | **-** | **-** | **-** | **-** |  |  |
| **Muscle degeneration** | + | + | + | - | **-** | **+** | **+** | **-** | **-** | **-** | **-** |  |  |
| **Intellectual disability** | | - | - | - | - | **-** | **+** | **+** | **-** | **-** | **+** | **+** | |
| **Self-care/ self-feed** | | + | - | + | + | **+** | **-** | **-** | **-** | **-** | **+** | **+** | |
| **Skeletal** | | Hammer toes | Hammer toes | Hammer toes &  Scoliosis | OFC 47.1cm | **-** | **-** | **-** | **-** | **-** | **-** | **-** | |
| **Gait** | | Abnormal gait | Abnormal gait | Abnormal gait |  |  |  |  |  |  |  |  | |
| **Movement** | | Ambulation difficulty | Ambulation difficulty | Ambulation difficulty | Walking with support | Walking with support | On buttocks | On buttocks | Sitting and walking delayed | Sitting and walking delayed | Sitting and walking  Delayed | Sitting and walking Delayed | |
| **Epilepsy** | | - | - | - | **-** | **-** | Fits, since 7 days old |  | Fit (with foaming) | every 2^-^3 days  (Stopped with medication) |  |  | |
| **Cardiac and Respiratory** | | Mild pulmonary stenosis  VSD (Heart) | Respiratory infections | Respiratory infections | **-** | **-** | - | - | Blood transfusion (9 bottles in past year) | Blood transfusion (4 bottles in past year) | - | - | |
| **Speech** | | Present | Present | Present | Present | Present | Absent | Absent | Delayed | Delayed | Present | Present | |
| **Other** | |  |  |  | C-section | C-section | Aggressive |  | High blood creatinine | High blood creatinine |  |  | |

**Supplementary Table 3: List of variants remaining after filtering (Family A)**

| **Chromosome** | **Position** | **rs ID** | **Ref seq** | **Alt seq** | **Gene** | **HGVS** | **Individual**  **143:2** | **Individual**  **143:3** | **PolyPhen2** | **CADD Score (v1.7)** | **Variant consequence** | **Variant Class** | **gnomADe SAS Allele Frequency** | **clinVar Clinical Significance** | **ACGM Classification** |
| --- | --- | --- | --- | --- | --- | --- | --- | --- | --- | --- | --- | --- | --- | --- | --- |
| Chr1 | 11711942 | rs143337024 | G | A | *DRAXIN* | NM_198545.4 c.734G>A,  p.(Gly245Asp) | A/A | A/A | Benign  (0.213) | 17.35 | Missense | SNV | 0.00323 |  |  |
| Chr1 | 56696099 | rs369893630 | C | T | *PRKAA2* | NM_006252.4 c.728C>T,  p.(Thr243Ile) | T/T | T/T | Benign (0.122) | 23.8 | Missense | SNV | 0.005977 |  |  |
| Chr1 | 12775917 | rs766228967 | G | C | *PRAMEF12* | NM_001080830.5  :c.662G>C:p.Arg221Thr | C/C | C/C | Benign  (0.001) | 0.001 | Missense | SNV | 0.0001391 |  |  |
| Chr16 | 55491906 | rs794727916 | AG | A | *MMP2* | NM_004530.6 c.1287del,  p.(Asn430ThrfsTer68) | A/A | A/A | N/A | 33.0 | Frameshift | DEL | 0.003266 | Pathogenic | Pathogenic |
| Chr18 | 54372468 | rs371851488 | C | A | *C18orf54* | NM_001288980.2 c.1329C>A,  p.(Ser443Arg) | A/A | A/A | Benign (0.084) | 16.79 | Missense | SNV | 0.0006143 |  |  |

**Supplementary Table 4: List of variants remaining after filtering (Family B)**

| **Chromosome** | **Position** | **rs ID** | **Ref seq** | **Alt seq** | **Gene** | **HGVS** | **Individual**  **30:1** | **Individual**  **30:2** | **PolyPhen2** | **CADD Score (v1.7)** | **Variant consequence** | **Variant Class** | **gnomADe SAS Allele Frequency** | **ClinVar Clinical Significance** | **ACGM Class** |
| --- | --- | --- | --- | --- | --- | --- | --- | --- | --- | --- | --- | --- | --- | --- | --- |
| Chr 4 | 989984 | rs558586992 | C | T | *SLC26A1* | NM_022042.4 c.955G>A, p.(Val319Met) | T/T | T/T | Probably Damaging (0.993) | 26 | Missense | SNV | 0.001016 |  |  |
| Chr 9 | 130885027 | rs756505258 | A | G | *ABL1* | NM_005157.6  c.2737A>G,  p.(Lys913Glu) | G/G | G/G | Benign (0.166) | 23.5 | Missense | SNV | 0.001927 |  |  |
| Chr 9 | 131519485 | N/A | A | G | *POMT1* | NM_001077365.2  c.1583A>G,  p.(Gln528Arg) | G/G | G/G | Probably Damaging (0.981) | 32 | Missense | SNV | 0.006699 | No clinical data available | Variant of uncertain significance |
| Chr 14 | 37796343 | rs559025860 | A | G | *TTC6* | NM_001310135.5 c.3841A>G,  p.(Ile1281Val) | G/G | G/G | Possibly Damaging (0.753) | 24.4 | Missense | SNV | 4.347e-05 |  |  |
| Chr 15 | 39807118 | N/A | G | A | *GPR176* | NM_007223.3  c.313C>T,  p.(Pro105Ser) | A/A | A/A | Benign  (0.35) | 23.3 | Missense | SNV | 0.0002289 |  |  |

**Supplementary Table 5: List of variants remaining after filtering (Family C)**

| **Chromosome** | **Position** | **rs ID** | **Ref seq** | **Alt seq** | **Gene** | **HGVS** | **Individual 25:1** | **Individual 25:2** | **PolyPhen2** | **CADD score (v1.7)** | **Variant Consequence** | **Variant class** | **gnomAD**  **SAS_AF** | **clinVar Clinical Significance** | **ACGM Class** |
| --- | --- | --- | --- | --- | --- | --- | --- | --- | --- | --- | --- | --- | --- | --- | --- |
| Chr 1 | 973588 | rs147671258 | C | T | *PLEKHN1* | NM_032129.3 c.1382C>T,  p.(Thr461Ile) | T/T | T/T | Probably Damaging (0.999) | 24.3 | Missense | SNV | 0.0008828 |  |  |
| Chr1 | 1641711 | rs1462211439 | CTCCTCT | C | *CDK11B* | NM_033486.3 c.954_959del,  p.(Glu322_Glu323del) | C/C | C/C | N/A | 15.02 | Inframe Deletion | Deletion | 0.005285 |  |  |
| Chr 1 | 5969232 | rs1557860870 | GGT | G | *NPHP4* | NM_015102.5 c.305_306del,  p.(Asn102ThrfsTer13) | G/G | G/G |  | 27 | Frameshift | Deletion | 0.00003875 | No clinical data available | Likely Pathogenic |
| Chr 1 | 15873082 | rs769694476 | GTCCCCC | G | *SPEN* | NM_015001.3 c.351_356del,  p.(Pro119_Pro120del) | G/G | G/G |  | 22 | Inframe Deletion | Deletion | 0.0001307 | No clinical data available | Variant of uncertain significance |
| Chr 8 | 37844879 | rs780832243 | G | A | *BRF2* | NM_018310.4 c.871C>T,  p.(Arg291Trp) | A/A | A/A | Possibly Damaging  (0.715) | 27.3 | Missense | SNV | 0.0007186 |  |  |
| Chr 9 | 35841614 | rs1025949651 | C | T | *TMEM8B* | NM_001042590.4 c.1129C>T,  p.(Arg377Cys) | T/T | T/T | Benign  (0.093) | 25.7 | Missense | SNV | N/A |  |  |
| Chr X | 50086427 | rs781967363 | C | T | *CLCN5* | NM_001127898.4 c.1114C>T,  p.(Arg372Cys) | T/T | T/T | Possibly Damaging (0.852) | 29.6 | Missense | SNV | 0.0005787 |  |  |

**Supplementary Table 6: List of variants remaining after filtering (Family D)**

| **Chromosome** | **Position** | **rs ID** | **Ref seq** | **Alt seq** | **Gene** | **HGVS** | **Individual 26:1** | **Individual 26:2** | **PolyPhen2** | **CADD score (v1.7)** | **Variant Consequence** | **Variant class** | **gnomADe SAS Allele Frequency** | **clinVar Clinical Significance** | **ACGM Class** |
| --- | --- | --- | --- | --- | --- | --- | --- | --- | --- | --- | --- | --- | --- | --- | --- |
| Chr 3 | 89449280 | rs73139269 | T | A | *EPHA3* | NM_005233.6 c.2402T>A,  p.(Phe801Tyr) | A/A | A/A | Probably Damaging (0.977) | 29.3 | Missense | SNV | 0.003038 |  |  |
| Chr 3 | 94036664 | rs764109067 | G | A | *ARL13B* | NM_001174150.2 c.599G>A,  p.(Arg200His) | A/A | A/A | Probably Damaging (0.977) | 33 | Missense | SNV | N/A | Pathogenic | Pathogenic |
| Chr 3 | 102468969 | rs533482871 | A | G | *ZPLD1* | NM_001329788.2 c.767A>G,  p.(Asp256Gly) | G/G | G/G | Benign (0.007) | 22.2 | Missense | SNV | 0.001757 |  |  |

**Supplementary Table 7: List of variants remaining after filtering (Family E)**

| **Chromosome** | **Position** | **rs ID** | **Ref seq** | **Alt seq** | **Gene** | **HGVS** | **Individual 50:3** | **Individual 50:5** | **PolyPhen2** | **CADD score (v1.7)** | **Variant Consequence** | **Variant class** | **gnomADe SAS**  **Allele Frequency** | **clinVar Clinical Significance** | **ACGM Class** |
| --- | --- | --- | --- | --- | --- | --- | --- | --- | --- | --- | --- | --- | --- | --- | --- |
| Chr 2 | 90005607 | G | G | A | *IGKV2D-24* | c.338G>A,  p.(Cys113Tyr) | A/A | A/A | Possibly Damaging (0.878) | 19.62 | Missense | SNV | 0.005681 |  |  |
| Chr 15 | 25716813 | G | G | T | *ATP10A* | NM_024490.4  c.1693C>A,  p.(Leu565Met) | T/T | T/T | Probably Damaging (0.956) | 23.2 | Missense | SNV | 0.00006676 |  |  |
| Chr 15 | 44566247 | CAG | CAG | C | *SPG11* | NM_025137.4  c.6811_6812del,  p.(Leu2271AspfsTer68) | C/C | C/C |  | 34 | Frameshift | Deletion | N/A | Pathogenic | Pathogenic |
| Chr 19 | 812946 | T | T | C | *PLPPR3* | NM_001270366.2  c.1781A>G,  p.(His594Arg) | C/C | C/C | Benign (0) | 17.84 | Missense | SNV | 0.0003015 |  |  |
| Che X | 151700692 | C | C | T | *PRRG3* | NM_001372163.1  c.355C>T,  p.(Arg119Trp) | T/T | T/T | Probably Damaging (0.98) | 27.5 | Missense | SNV | 0.0008473 |  |  |

**Supplementary Figure 1: Map showing the location of five families sampled from different region of Pakistan**

**
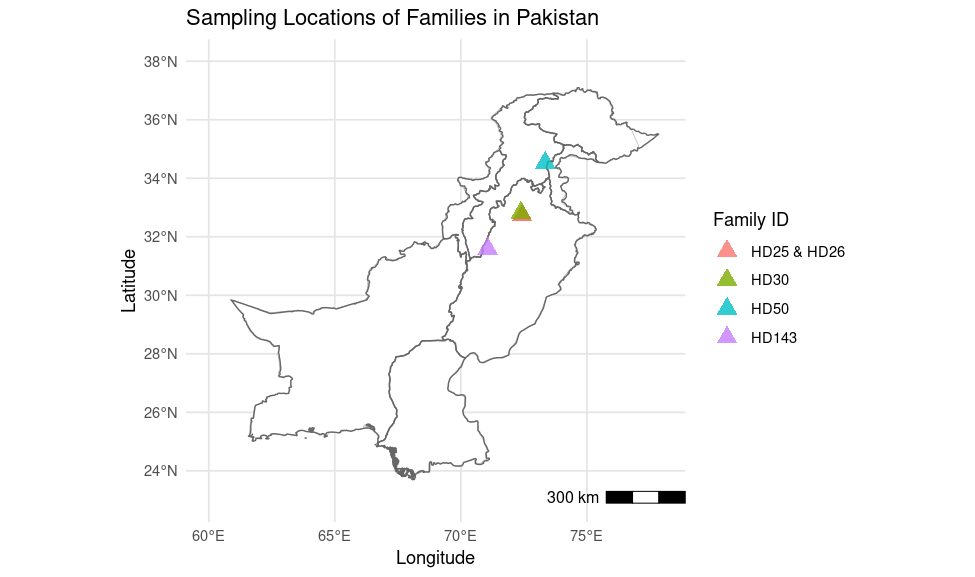
**


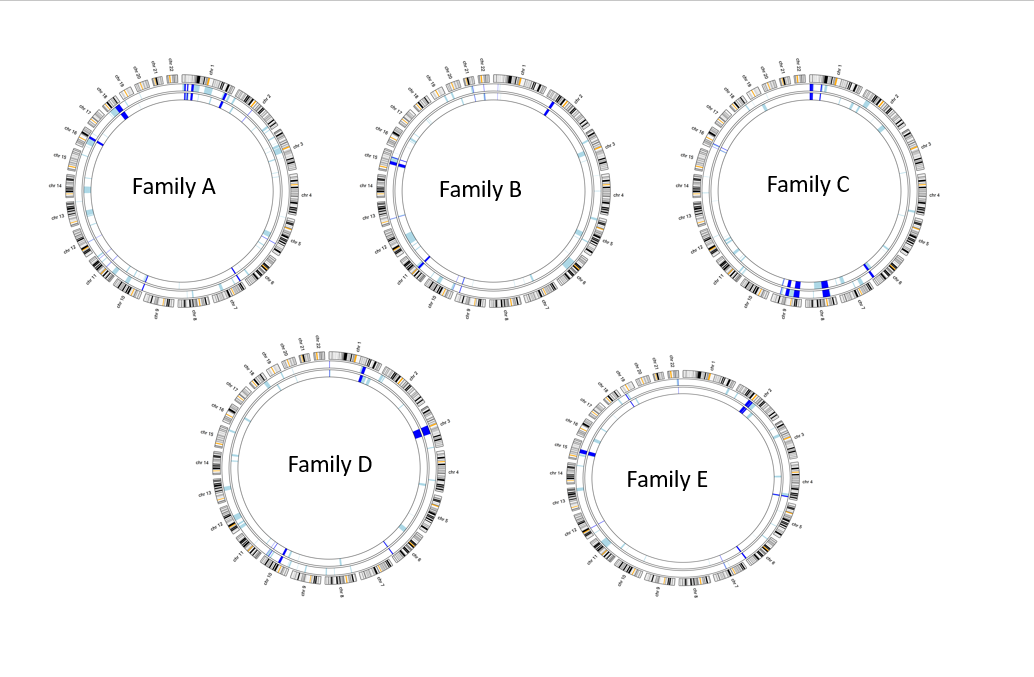
**Supplementary Figure 2: Ideograms showing regions of homozygosity shared between affected individuals with exome data for each Family**
